# Jacalin capped platinum nanoparticles confer persistent immunity against multiple *Aeromonas* infection in zebrafish

**DOI:** 10.1038/s41598-018-20627-3

**Published:** 2018-02-02

**Authors:** Khan Behlol Ayaz Ahmed, Thiagarajan Raman, Anbazhagan Veerappan

**Affiliations:** 1 School of Chemical and Biotechnology, SASTRA University, Thirumalaisamudram, Thanjavur, 613401 Tamil Nadu India; 2 Department of Advanced Zoology and Biotechnology, Ramakrishna Mission Vivekananda College, Mylapore, Chennai 600004 India

## Abstract

Bacterial resistance is a major clinical problem, which is compounded by both a lack of new antibiotics and emergence of multi- and extremely-drug resistant microbes. In this context, non-toxic nanoparticles could play an important role in conferring protection against bacterial infections and in this study we have made an attempt to show the usefulness of jacalin capped platinum nanoparticles in protecting zebrafish against multiple infections with *Aeromonas hydrophila*. Our results also indicate that use of nanoparticles promotes adaptive immune response against the pathogen, so much so that zebrafish is able to survive repetitive infection even after twenty one days of being treated with jacalin-capped platinum nanoparticles. This is significant given that platinum salt is not antibacterial and jacalin is non-immunogenic. Our study for the first time reveals a novel mechanism of action of nanoparticles, which could form an alternate antibacterial strategy with minimal bacterial resistance.

## Introduction

Immune system plays a vital role in surviving common bacterial infection^1,2^, however, it alone is insufficient to curtail severe infections. Antibiotics, which function to reduce the spread or eliminate microbe have been successfully used to save millions of lives^3^. Unfortunately, overuse of antibiotics has led to emergence of antibiotic-resistant bacteria^4^. The mode of action of antibiotics itself was suggested as a reason for resistance development^5^. Due to resistance, conventional antibiotic therapies fail causing mortality and thus developing new therapeutic modality is important. Strategically, boosting the immune system is a good option^6^, because it has several advantages such as: (i) the drug will not act on bacteria directly, so chance of rapid emergence of resistance will be reduced and (ii) the method can also be used to treat bacterial infection in immune-compromised patients.

Developments in nanotechnology have revealed metal nanoparticles as promising new generation antibacterial agents^7–9^. However, the mechanism of action of nanoparticles remains enigmatic. It is proposed that the NPs can disrupt bacterial membrane integrity, generate reactive oxygen species and result in bacterial cell damage^10,11^. In spite of extensive developments towards the understanding of antibacterial action of NPs, their influence on the host immune system is largely unknown. In general, immune system responds to invading pathogens and also to antibacterial agents in a rapid, efficient and self-limiting manner^12–14^. An appropriate stimulation of immune response after infection is crucial to successful treatment of infection with NPs. So far, such knowledge is unavailable with metal nanoparticles.

Based on the available literature, we were convinced that platinum nanoparticles (PtNPs) were less toxic with good antibacterial activity^15–19^. Based on *in vitro* study, it was proposed that PtNPs kills bacteria through disruption of the cell envelope and through the production of reactive oxygen species^19^. Although, we showed that PtNPs rescue zebrafish from bacterial infection, however, *in vivo* mechanism of action remains unclear. To understand this, we prepared jacalin capped platinum nanoparticle (JPtNPs) and evaluated immune response of infected zebrafish during the course of treatment with JPtNPs. The choice of jacalin is made because it is a non-immunogenic protein, isolated from the seeds of *Artocarpus integrifolia*^20^. Jacalin displays broad specificity to various glycans, including galactose, *N*-acetylygalactosamine, mannose, glucose, *N*-acetylmuramic acid and *N*-acetylneruamic acid^21^. Since the bacterial cell wall is composed of a wide array of glycans and forms a major protective barrier for a bacterium, it naturally becomes a critical target for any new antibiotics^22^. Our study for the first time showed that JPtNPs exert antibacterial activity by influencing the immune response of the host to suppress bacterial infection.

## Result and Discussion

### Preparation of jacalin capped platinum nanoparticles

**Figure 1:**
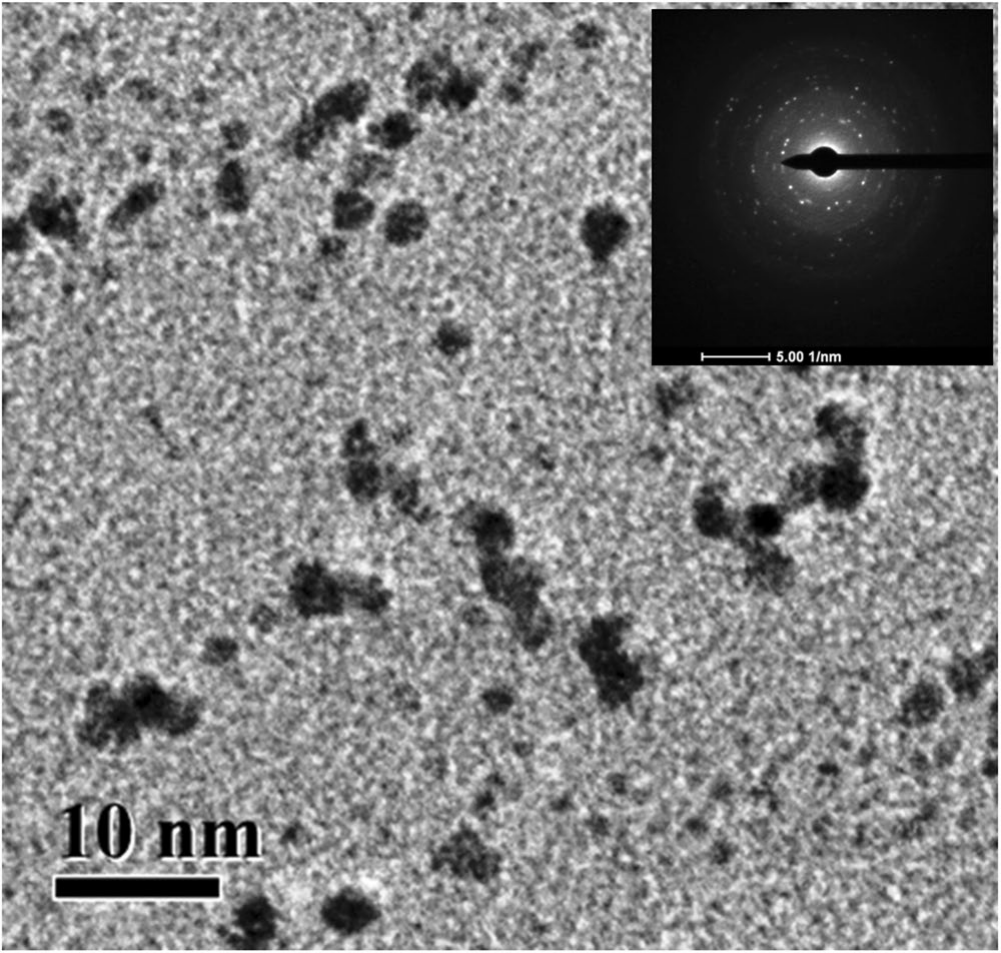
(A) TEM micrograph of JPtNPs. Inset corresponds to the SAED pattern.

Typically, chloroplatinic acid (0.5 mM) on reduction with NaBH_4_ in the presence of jacalin produces JPtNPs. Formation of elemental platinum was confirmed by energy dispersive X-ray spectroscopy (Fig. S1). Atomic absorption spectroscopy confirmed the concentration of prepared JPtNPs as 0.505 ± 0.04 mM. Functionalization of PtNPs with jacalin was verified with FTIR (Fig. S2). Transmission electron microscopy reveals that JPtNPs are ~3.1 ± 1.6 nm in diameter (Fig. 1). The observed rings in the selected area electron diffraction (SAED) patterns confirm the crystalline nature of JPtNPs (inset of Fig. 1). Particle size analysis showed increased particle size (72.57 nm) due to hydration of capping agent with polydispersity index of 0.221 (Fig. S3). This suggests that PtNPs are relatively monodispersed and reside in the nanoscopic template of jacalin. The zeta potential value of −23.0 mV confirms that the electrical boundaries of PtNPs are well separated and prevented the PtNPs from agglomeration (Fig. S4).

### *In vitro* antibacterial activity

The aim of the work was to test JPtNPs against zebrafish infection model. Thus, to start with, antibacterial study with JPtNPs was commenced against fish specific Gram-negative pathogen, *A. hydrophila*. The minimum inhibitory concentration (MIC) of JPtNPs was determined using resazurin microtitre plate assay. MIC was observed to be 31.25 µM against *A. hydrophila* (Fig. S5). The zone of inhibition (ZOI) was observed around the wells loaded with JPtNPs, suggesting its antibacterial activity (Fig. S6A). Bacterial colony count assay revealed that JPtNPs were able to completely inhibit *A. hydrophila* proliferation (Fig. S6B–G). JPtNPs also displayed ZOI against various Gram-negative and Gram-positive bacterial strains (Fig. S7), indicating a broader antibacterial activity. Noteworthy that jacalin and the NPs precursor chloroplatinic acid have no antibacterial activity. In order to understand the significance of jacalin, we prepared PtNPs with pectin instead of jacalin^19^. Pectin capped PtNPs (pPtNPs) was mixed with jacalin and tested their antibacterial activity by bacterial colony forming assay. LB agar plates containing pPtNPs showed higher number bacterial colonies than the mixture of jacalin and pPtNPs (Fig. S8). Strikingly, the number of colonies formed in JPtNPs is comparable to the mixture of jacalin and pPtNPs (Fig. S8). These results suggest that the presence of jacalin either as capping agent or complex with NPs can enhance the antibacterial activity.

**Figure 2:**
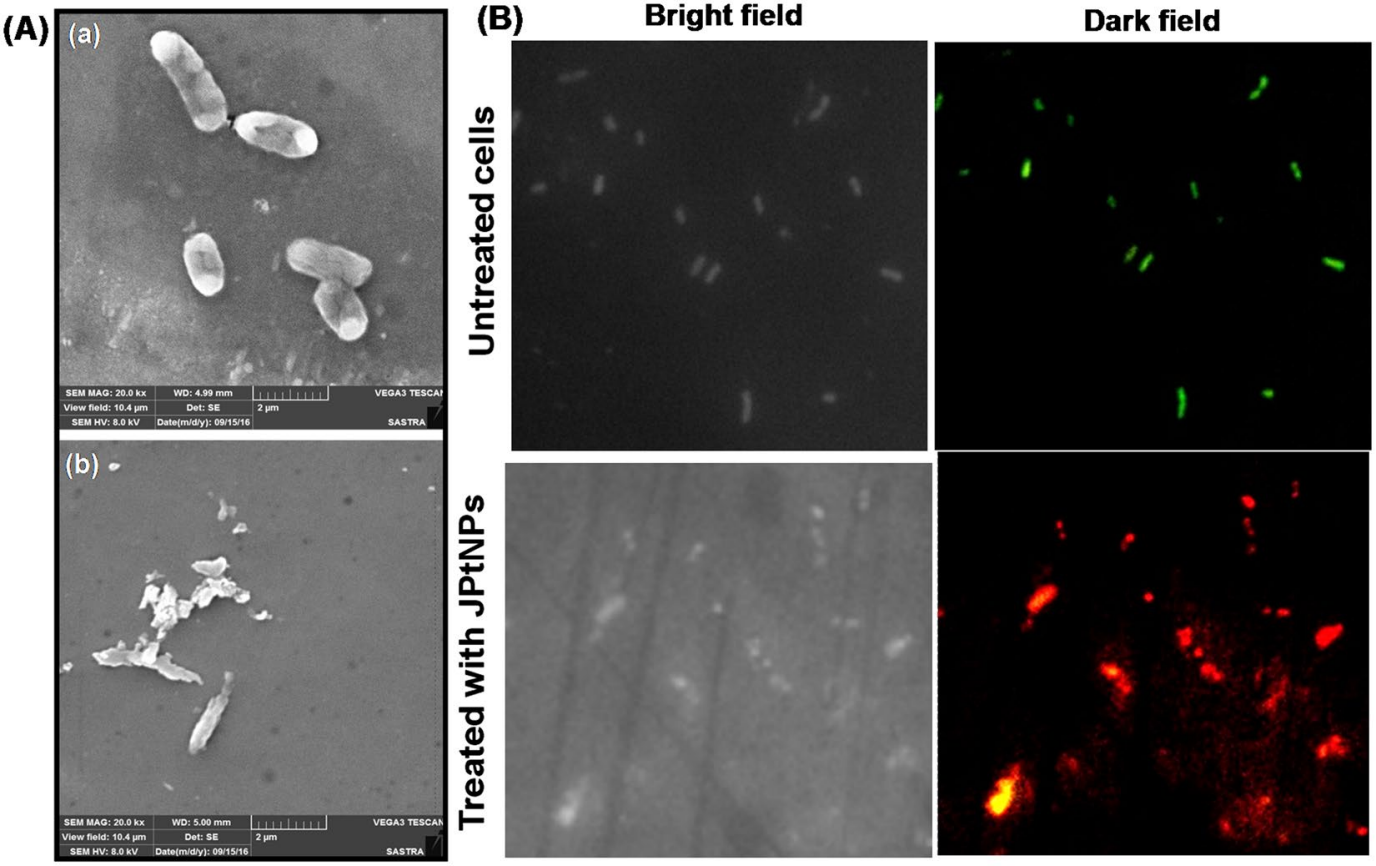
Membrane integrity study. (**A**) Scanning electron micrograph of *A. hydrophila* (a) untreated and (b) treated with 50 µM JPtNPs. Bacterial cells treated with JPtNPs showed morphological changes of cell membrane. (**B**) Fluorescence microscopy image of cells stained with PI/AO. *A. hydrophila* cells were stained after 12 h incubation with 50 µM JPtNPs. Dark field image revealed that cells treated with JPtNPs have compromised membranes.

*A. hydrophila* morphology without JPtNPs treatment exhibited intact cell membrane (Fig. 2A). However, cells treated with JPtNPs showd dramatic change in bacterial morphology, suggesting loss of membrane integrity. This irreversible alteration in morphology is attributed to the interaction between JPtNPs and bacterial membrane, resulting in membrane damage as evidenced by bacterial disintegration (Fig. 2A). The membrane integrity was further investigated by propidium iodide (PI)/acridine orange (AO) dual staining. The untreated cells showed green fluorescence, whereas the cells treated with JPtNPs display red fluorescence, indicating loss of bacterial membrane integrity (Fig. 2B). Interestingly, human red blood cells treated with 250 µM JPtNPs (8 times higher than MIC) showed morphology similar to the control (Fig. S9), indicating that JPtNPs is hemocompatible without any toxicity.

### *In vivo* antibacterial activity

Having observed good antibacterial activity, we decided to test the efficacy of JPtNPs against *A. hydrophila* infected zebrafish. About 20 healthy fish were intramuscularly infected by injecting 10 µL (0.1 OD_660 nm_) *A. hydrophila* and divided into groups. Group-A served as infected control. After three hours, Group-B infected fish were treated by injecting 10 µL of JPtNPs (50 µM). Group-A fish succumbed to infection within 8 h while group-B remained alive and appeared similar to the uninfected fish (Group-C). The present results are better than the previous report because here we use only one dose of 50 µM JPtNPs to rescue fish from infection, whereas in the case of pectin capped PtNPs three doses of 100 µM NPs is required^19^. This indicates that jacalin plays an important role in improving the efficacy of PtNPs.

**Figure 3:**
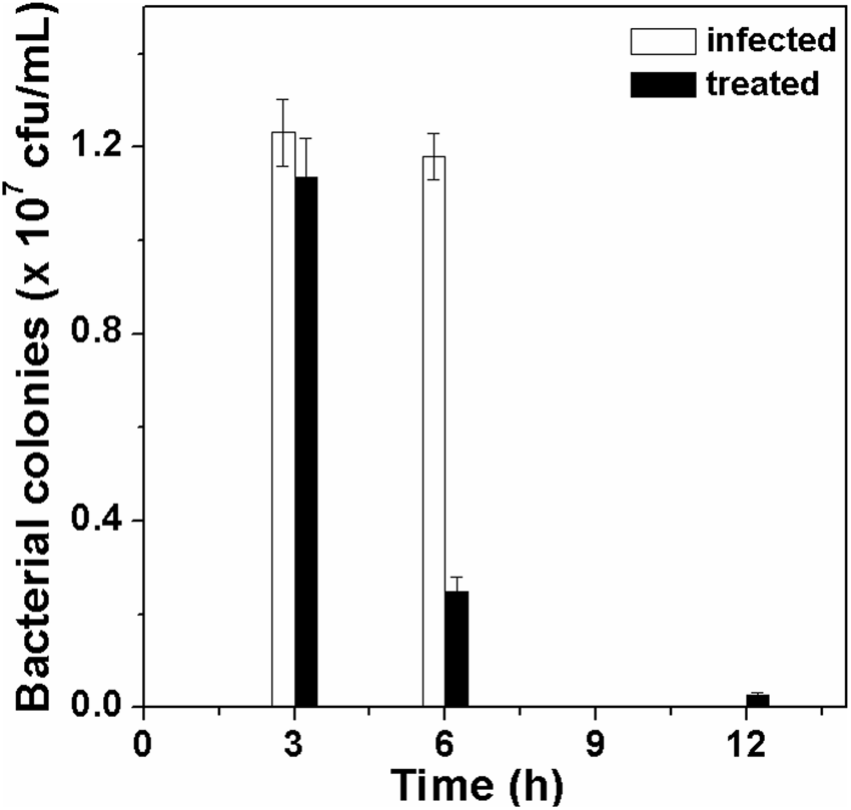
*In vivo* antibacterial activity. Number of viable cells present in the zebrafish at different time points.

The infection levels in each group were determined by scarifying a fish at definite time interval, and muscle (approx. 100 mg) were dissected and homogenized in PBS. The homogenate was serially diluted (dilution factor-10^−4^) and plated on a LB agar plate and cultured for 24 h at 37 °C (Fig. S10). Group-A fish showed higher bacterial numbers at 3 h, which went on to increase resulting in mortality by 8 h (Fig. 3). On the other hand, group-B fish showed a marked decrease in bacterial numbers at 6 h and 12 h. As a result, all the fish completely recovered from infection and were normal.

Next, the ability of the JPtNPs treated group-B fish to withstand repeated bacterial infection was studied. First, the fish were infected (first infection) and treated successfully as mentioned above. After seven days, the treated fish were re-infected (second infection) with *A. hydrophila*, but these fish were not treated again with JPtNPs. Strikingly, all the re-infected fish survived. Next, again after a span of seven days, the same set of fish was infected (third infection) with *A. hydrophila*. Remarkably, the fish survived the third infection too. These results suggest that JPtNPs administration to one time infected fish enable the same fish to survive multiple infections with the same pathogen. However, it is to be noted here that if JPtNPs are administered before any infection, then it fails to confer protection.

### Immunomodulatory effect of JPtNPs

**Figure 4:**
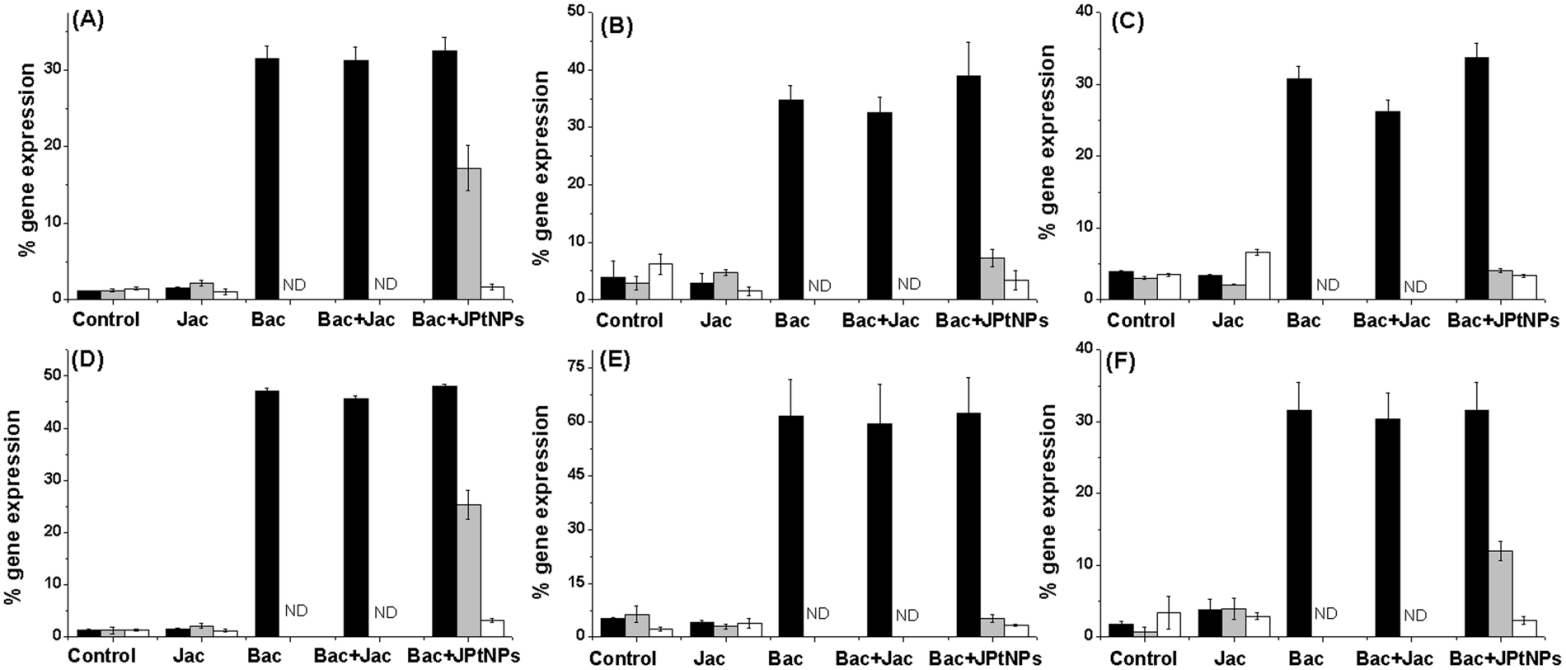
Modulation of cytokine gene expression by JPtNPs in infected zebrafish.The various groups are: Control − uninfected zebrafish; Jac − zebrafish injected with jacalin alone; Bac − zebrafish infected with *A. hydrophila* alone; Bac + Jac − zebrafish infected with *A. hydrophila* and then treated with jacalin; Bac + JPtNPs − zebrafish infected with *A. hydrophila* and treated with JPtNPs. The concentrations of the various compounds are as mentioned in the methods section. The genes analyzed are (**A**) TLR-4a; (**B**) IL-1β, (**C**) TNF-α, (**D**) IFN-γ, (**E**) iNOS, and (**F**) IL-4. The colour of the bar represents the different time points at which gene expression was monitored: Black bar −3 h; gray bar −6 h and white bar −12 h. Bars indicate mean ±SD of five determinations where the samples were run in duplicates.

To test if JPtNPs modulate immune related genes to exert the prolonged antibacterial activity against *A. hydrophila*, we went on to analyze changes in cytokine gene expression. Cytokines are important for setting up inflammatory cascade leading to recruitment and activation of immune cells at the site of infection. The following genes were analyzed: TLR-4A, proinflammatory IL-1β, TNF-α, IFN-γ, iNOS and anti-inflammatory IL-4. Infection of zebrafish with *A. hydrophila* resulted in significant enhancement in gene expression of TLR-4A after 3 h of infection (36-fold), when compared to uninfected or jacalin alone treated fish (1.86-fold) (Fig. 4A). As expected this increase in TLR-4A gene was prominent in all groups of fish that had been infected with bacteria and this suggests the importance of TLR-4A in recognition of pathogen associated molecular patterns (PAMPs) on *A. hydrophila*. This initial recognition of PAMPs is necessary for establishing successful immunity^23,24^. However, when TLR-4A gene expression was analyzed at different time points, (6 h and 12 h), it was not at all observed in bacteria infected group and infected fish treated with jacalin alone. The absence of TLR-4A gene expression in these two groups could have contributed to failure of immune recognition and thus resulting in establishment of infection. However, in the case of zebrafish that were treated with JPtNPs, TLR-4A levels were observed to be significantly elevated (6 h: 13.66-fold and 12 h: 2.5-fold) and this could be important for ‘persistent’ recognition of *A. hydrophila*, and thus enabling an effective immune response. This enhanced expression of TLR-4A in infected zebrafish treated with JPtNPs can be correlated to inflammation. Inflammation onset is important for reducing the spread of infectious agents^25^ and also for recruitment and activation of immune cells^26–28^. Our results shown that infection of zebrafish with *A. hydrophila* resulted in significant enhancement, (3 h post infection), in the expression of pro-inflammatory cytokines namely IL-1β (11.3-fold), IFN-γ (34-fold), TNF-α (10.24-fold) and iNOS (12.92-fold) (Fig. 4B–E). However, when the response of these cytokines and iNOS were checked at 6 h and 12 h post infection, the levels were far too low to be detected in infected zebrafish that were either untreated or treated with jacalin alone (Fig. 4E). Interestingly, expression of all the pro-inflammatory genes was observed at 6 h and 12 h post-infection in case of zebrafish that were treated with JPtNPs (Fig. 4B–E). Among the cytokines, IFN-γ gene expression was observed to be significantly enhanced at 6 h post infection (19.01-fold) (Fig. 4D). These results clearly show the ability of JPtNPs in producing immune modulation in fish infected with bacteria. What is important is that the level of expression of these pro-inflammatory genes could be related to that of TLR-4A genes. Thus, by enhancing TLR-4A gene expression, JPtNPs are able to stimulate pro-inflammatory cytokines, which could be beneficial in terms of host resistance against bacterial infection^29^. While the absence of any TLR-4A or pro-inflammatory gene expression 6 h or 12 h post infection in infected zebrafish either left untreated or jacalin treated, correlates well with the susceptibility of zebrafish to *A. hydrophila*. Unexpectedly, when gene expression of anti-inflammatory IL-4 was analyzed, it showed a similar trend like that of pro-inflammatory cytokines across different treatment groups (Fig. 4F). Treatment of infected zebrafish with JPtNPs produced high levels of IL-4 (18.05-fold for 6 h and 6.28-fold for 12 h). Though, studies have shown the ability of pathogens to stimulate anti-inflammatory cytokines^30^ as an immune escape strategy, we consider that increase in IL-4 might be important for subsequent antibody responses, during late stages of infection^31,32^.

### Immunebooster activity of JPtNPs

**Figure 5:**
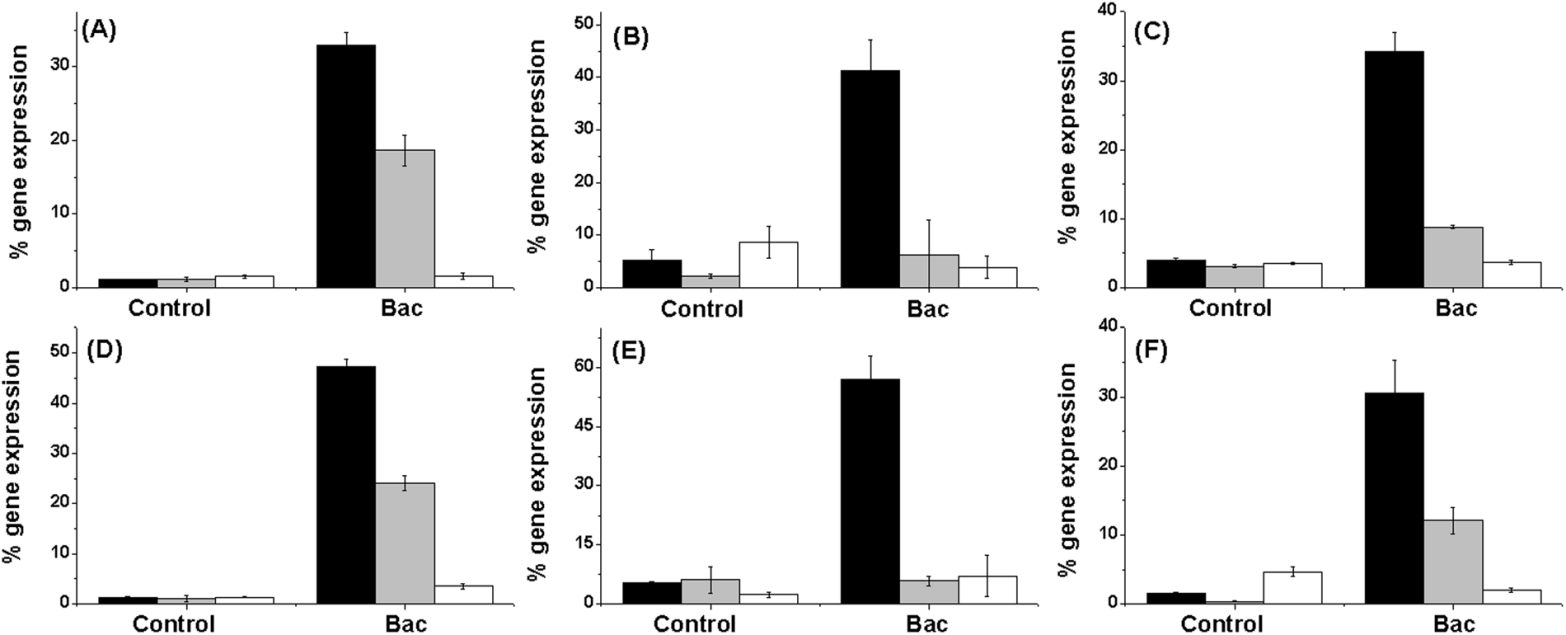
Resensitization of zebrafish to second *A. hydrophila* infection by JPtNPs.The groups are: Control – no second infection, Bac – zebrafish infected for the second time with *A. hydrophila*. Note: Both control and Bac group fishes were previously rescued from *A. hydrophila* infection by JPtNPs. The genes analysed are (**A**) TLR-4A, (**B**) IL-1β, (**C**) TNF-α, (**D**) IFN-γ, (**E**) iNOS, and (**F**) IL-4. The colour of the bar represents the different time points at which gene expression was monitored: Black −3 h; gray −6 h and white −12 h. Values are mean ± SD of five determinations using samples from different preparations and run in duplicates.

To give evidence to the fact that JPtNPs are highly immunomodulatory and are beneficial in terms of repeat infections too, we performed a series of re-sensitization experiments. For this, zebrafish, which were previously rescued from *A. hydrophila* infection using JPtNPs were re-infected with *A. hydrophila* after 21 days. However, this time the re-infected fish were left untreated and cytokine expression was analyzed. As shown in Fig. 5, re-sensitization with *A. hydrophila* resulted in significantly enhanced TLR-4A expression after 3 h (31-fold) and 6 h (15-fold) post infection when compared to non re-sensitized control. Similar to TLR-4A, levels of pro inflammatory TNF-α (10.25-fold), iNOS (15-fold), IL-1β (31-fold), and IFN-γ (13.86-fold) were significantly enhanced after 3 h of re-sensitization (Fig. 5B–E). Among these, IFN-γ (2.5-fold), IL-1β (18-fold), and TNF-α (3.11-fold) were significantly enhanced even 6 h after re-sensitization, when compared to their respective controls. Similarly, IL-4 (21-fold) was found to be significantly enhanced 3 h after re-sensitization. These re-sensitization experiments suggest that treatment with JPtNPs appears to produce not only a strong but also a persistent immunomodulation.

Cytokines secreted by inflamed tissues are essential in modulating host immune response to a pathogen^33–36^. Thus, IL-1β increase upon infection by bacteria appears to be an acute phase response^37^, whereas an increase in TNF-α, along with IL-1β, would contribute to the recruitment of phagocytic cells to the site of infection^38^ and their subsequent activation. IFN-γ is another pro-inflammatory cytokine vital for Th1 responses^39^ and their levels were elevated in our study. Together with re-sensitization experiments it is clear that infection with *A. hydrophila* can result in mortality of untreated zebrafish. Upon treatment with JPtNPs, there is an increase in TLR-4A expression on cell surface, which results in activation of pro-inflammatory mediators such as TNF-α, IFN-γ and IL-1β. One consequence of this enhancement is the increase in expression of iNOS gene^40^ that contributes to pathogen killing^41^.

In re-sensitization experiments we observed elevated levels of IL-4, both upon infection and upon treatment of infected zebrafish with JPtNPs. This is an interesting observation, since IL-4 is vital for promoting Th2 responses^42–44^ and is equally important for B cell growth and development^45^. However, IL-4 is anti-inflammatory and is well known to inhibit the production of pro-inflammatory cytokines such as IL-1β and TNF-α^43^ and antagonizes IFN-γ^46^.

### Antibody production triggered by JPtNPs

**Figure 6:**
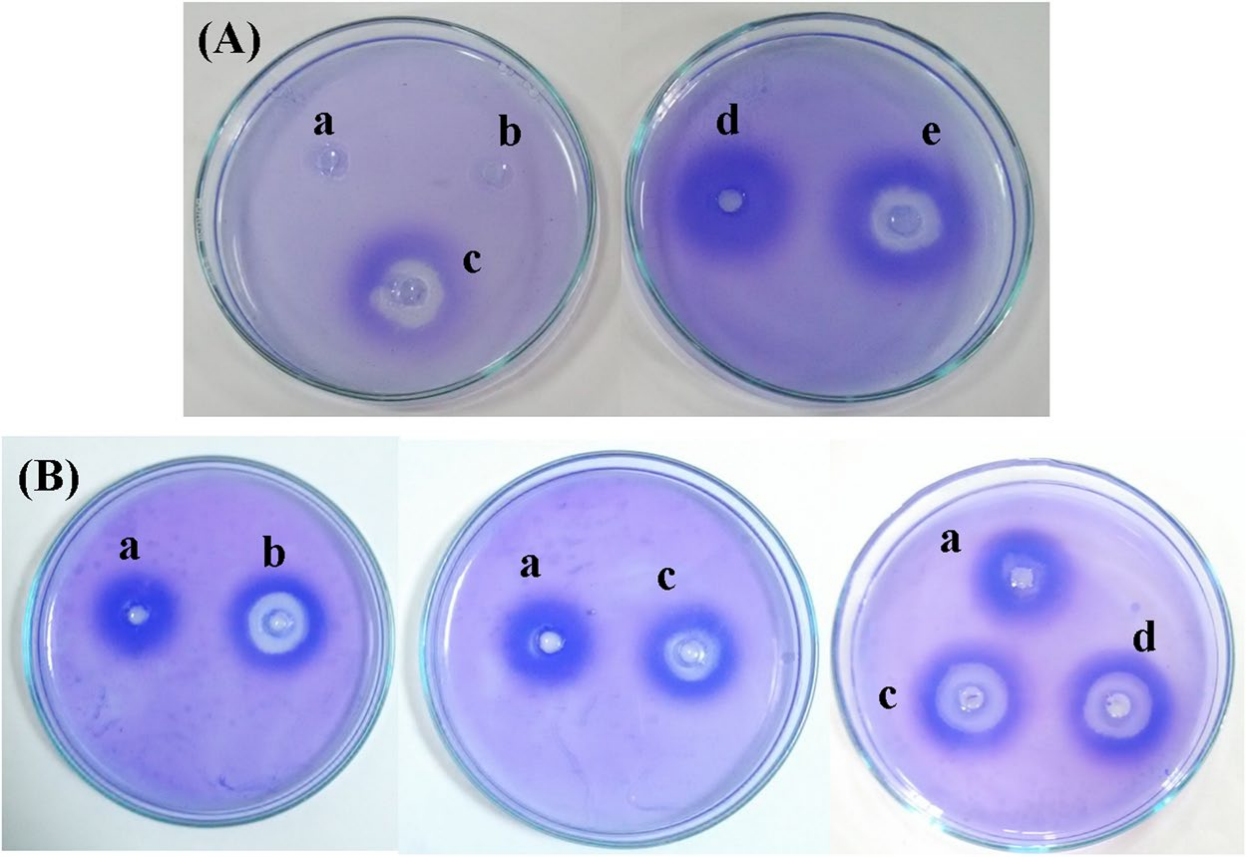
(**A**) Radial immuno assay. Serum from (i) uninfected control, (ii) control group-treated with jacalin alone, (iii) infected with *A. hydrophila* and treated with jacalin, (iv) infected with *A. hydrophila* alone, (v) infected with *A. hydrophila* and treated with JPtNPs. (**B**) Re-sensitization radial immuno assay. Serum collected from: (i) infected control, (ii) rescued zebrafish given second infection after three days, (iii) rescued zebrafish given third infection on day seven, (iv) third time infected and rescued fish after 21 days and (v) rescued fish from the third time infection was infected again for the fourth time on day 21. Representative result of assay repeated thrice with different samples. Rescued zebrafish refers to infected and JPtNPs treated animal and this is the first infection.

On the basis of re-sensitization experiments, and enhanced IL-4 gene expression, we assumed that modulation of cytokine gene expression alone would be insufficient for combating bacterial infection. Thus, we surmised that JPtNPs could also facilitate antibody generation. To verify this hypothesis, we performed radial immuno assay (RIA) using heat killed *A. hydrophila* and anti-serum from zebrafish. In the first set of experiments anti-serum was collected from infected fish after 7 h, since by 8 h post infection with *A. hydrophila* there was 100% mortality. On the other hand, the anti-serum of infected zebrafish that were treated with JPtNPs was collected after 12 h. It was observed that control and jacalin alone treated fish showed no precipitation (Fig. 6Ai and ii), however, infected zebrafish treated with jacalin alone (12 h anti-serum) showed a moderate precipitation reaction (Fig. 6Aiii), but these fish did not withstand infection. Conversely, infected, but untreated fish (7 h anti-serum) (Fig. 6Aiv) and infected fish treated with JPtNPs (12 h anti-serum) showed strong precipitation reaction (Fig. 6Av). The infected, but untreated fish show diffuse zone, (Fig. 6Aiv), whereas the fish treated with JPtNPs showed very prominent and sharp zone (Fig. 6Av), indicating antibody production in zebrafish. Nevertheless, it is clear that pathogen-specific antibody production might not commence so early after infection, and thus the observed RIA reaction could be attributed to pre-formed antibodies with ‘affinity’ to *A. hydrophila* or remnants of response to a previous infection. Strikingly, there is no reaction for control (Fig. 6Ai) or jacalin alone (Fig. 6Aii) or JPtNPs alone treated groups (Fig. 6Aiii). These results suggest the possibility of *A. hydrophila* specific antibody generation. To confirm this claim, we re-infected the treated Group B fish with *E. coli* and found that fish succumbed to infection, unlike *A. hydrophila* infection.

To lend evidence to this, RIA was performed with samples after re-sensitization. For this we took infected zebrafish that were rescued with JPtNPs and divided them randomly into two groups. In the first group, the zebrafish were not re-infected with *A. hydrophila* and in the second group the zebrafish were re-infected with *A. hydrophila* on day 3, 7 and 21. For both groups the anti-serum was collected after 3, 7, and 21 days and RIA were performed. In addition, in the second group after 21 days, anti-serum was collected from fish before (Fig. 6Biv) and after infection (Fig. 6Bv) with *A. hydrophila*. Interestingly, anti-serum from the first group showed precipitation reaction after 3, 7, and 21 days (Fig. 6B-ii, iii and iv) and all these reactions were more or else similar. The zone was very diffuse. On the other hand, the precipitation zone observed with the second group of zebrafish (Fig. 6B-ii,iii and iv) was very sharp and bigger for all 3 samplings than that observed with the first group (Fig. 6Bi). A similar kind of zone was observed with zebrafish from second group after 21 days when anti-serum was collected before the infection (Fig. 6Biv). Together with the earlier RIA results, these observations show that there is a clear evidence for *A. hydrophila* specific antibody in the zebrafish. Such antibody production against *A. hydrophila* has been previously demonstrated in fish^47^. This is also supported by the fact that during re-sensitization, zebrafish was treated only once with JPtNPs while being infected multiple times with *A. hydrophila* and they survived. These results suggest that JPtNPs are very efficient in treating multiple bacterial infections by not only modulating pro-inflammatory cytokines but also promoting *A. hydrophila-*specific antibody responses. However, our study suffers from three limitations; i) antigen or antibody used in RIA was not purified, ii) the kinetics of JPtNP-induced antibody production is less clear and, iii) protein levels of the cytokines need to be measured. Nevertheless, this study clearly shows a novel mechanism of action of JPtNPs in combating bacterial infection in zebrafish by immunomodulation, and such a response has not been demonstrated for any nanoparticle so far. Hence, there is immense potential for the further development and use of such nanoparticles-based antibacterial strategies against highly resistant bacterial pathogens of humans.

## Materials and Methods

### Synthesis and characterization of jacalin capped platinum nanoparticles

Typically, 0.5 mM of hexachloroplatinic acid hexahydrate and 5 μM of jacalin was dissolved in 10 mL of double distilled water and mixed in the magnetic stirrer operating at the speed of 500 rpm. After 30 min, 10 mM sodium borohydride was added and the solution was stirred for 5 min and then allowed the reaction to proceed at room temperature for 6 h. The formation of jacalin capped platinum nanoparticles (JPtNPs) was evidenced from the appearance of brownish black colour. The obtained NPs were used as such in further studies. Size and crystallinity of JPtNPs was measured by transmission electron microscope (TEM). Samples for TEM were prepared by placing a drop of NPs solution on the copper grid and drying it in vacuum. TEM were taken in JEOL JEM-2100F, Japan operated at an accelerated voltage of 200 kV. The FT-IR spectra were recorded in a PerkinElmer FT-IR spectrometer with 1 cm^−1^ resolution. Jacalin and JPtNPs were freeze dried in a lyphophilizer and ground with KBr to obtain pellet for recording FT-IR. The presence of elemental platinum was measured by energy dispersive X-ray spectroscopy (EDAX). Zeta potential and particle size of the as-prepared JPtNPs was determined by a Malvern Zetasizer version 6.20.

### *In vitro* antibacterial activity of JPtNPs

Minimum inhibitory concentration (MIC) was determined by resazurin microtiter (REMA) plate method. The antibacterial activity of JPtNPs was further tested by well diffusion method against the following pathogenic bacteria: *E. coli* (MTCC723)*, P. aeruginosa* (MTCC1688)*, S. aureus* (MTCC3160)*, B. subtilis* (MTCC441), *A.hydrophilia, V. cholerae* (MTCC3904*), K. Pneumoniae* (MTCC109), *B. thurengensis* (MTCC869), *S. typhi* (MTCC98), *Shigella* (MTCC1457), *P. vulgaris* (MTCC7299). The zone of inhibition of bacteria was measured after incubation for 24 h at 37 °C. The detail procedure for *in vitro* antibacterial activity was given in supporting information.

### Bacterial membrane damage analysis

The membrane damage caused by JPtNPs in bacteria was initially assessed by scanning electron microscopy. *A. hydrophilia* was cultured in LB media in absence and presence of JPtNPs. After 12 h the culture was fixed by 2.5% glutaraldehyde for 1 h followed by washing with PBS buffer. Later the sample was dehydrated with ethanol and air dried on a small glass slide. The sample was analysed using TESCAN SEM.

### Bacterial membrane integrity study

The extent of membrane damage in bacteria caused by JPtNPs was evaluated by propidium iodide (PI) and acridine orange (AO) dual staining. Briefly, cells were grown to mid-log phase, in presence of JPtNPs and then collected and washed twice with PBS buffer and resuspended in an equal volume of the same buffer. AO/PI at a concentration of 0.5 mM was added to the bacterial culture and this was followed by JPtNPs. Bacterial cells untreated with JPtNPs were maintained as control. Fluorescence microscopy image was recorded using NIKON Eclipse Fluorescence Microscope after 2 h of incubation at 37 °C. FITC (green) filter was used for AO and PI (red) filter was used for visualizing live and dead cells respectively.

### *In vivo* antibacterial activity of Jac-PtNPs

All experiments were performed in compliance with the CPCSEA guidelines for laboratory animal facilities (Central Act 26 of 1982) and also followed approved institutional guidelines as prescribed by the Institutional Animal Ethics Committee (CPCSEA-493/SASTRA/IAEC/RPP) of SASTRA University, India. Optimizations of bacterial dosage to zebrafish for establishment of infection are detailed in supporting information. Infection was initiated intramuscularly and treatment was given from the opposite side to that of the infection. The *in vivo* bacterial infection and the treatment with JPtNPs was performed using zebrafish that were divided into 5 groups namely; Group A: Bacteria infected, Group B: Bacterial infected + treated with JPtNPs, Group C: Control, uninfected fish, Group D: uninfected fish + treated with jacalin, Group E: uninfected fish + treated with JPtNs. Each group consisted of 20 fish. In a typical experiment, 10 µl of 0.1 OD culture of *A. hydrophila* was injected into Group A and Group B, whereas Group D and Group D were injected with jacalin and JPtNPs, respectively. The fish were fed normally and allowed to rest for a period of 3 h. After 3 h of infection, 10 µl of 50 µM JPtNPs was injected into Group B. The fish were monitored for mortality due to bacterial infection for a period of 24 h. At regular time points, (3 h, 6 h, 12 h) one fish from each group was removed, anesthetized and sacrificed. Approximately 100 mg of muscle tissue was dissected and homogenized using sterile PBS. The homogenate was serially diluted using sterile PBS and plated on sterile LB agar plates. The plates were incubated for 24 h at 37 °C. After 24 h, bacterial colonies were counted and reported. All the experiments were performed in triplicates.

### Gene Expression Analysis

Total RNA was isolated from zebrafish using TRIzol. The purity and concentration of total RNA was checked using Nanodrop and absorbance at 260/280 calculated. The RNA sample was also checked for purity by running in a 1.2% agarose gel. Total RNA was then converted to cDNA using cDNA conversion kit (Sisco Research Laboratory). List and sequence of oligonucleotide primers used in this study were given in Table 1. Gene expression studies were done at different time points of 3 h, 6 h, 12 h in accordance to the antibacterial studies. β-actin, the housekeeping gene transcript was used to normalize the results by eliminating variations in mRNA and cDNA quantity and quality, and each mRNA level was expressed as ratio to β-actin mRNA. The PCR conditions used were as follows: denaturation for 30 s at 94 °C, 40 cycles of 30 s at 94 °C, 60 °C- 40 s, 72 °C − 40 s, followed by final extension at 72 °C for 10 min. At the end, the samples were analysed in 10% agarose gel and band density analysed using Quantity one densitometer. β-actin, the housekeeping gene transcript was used to normalize the results by eliminating variations in mRNA and cDNA quantity and quality, and each mRNA level was expressed as ratio to β-actin mRNA. PCR was run in duplicates and five independent runs were performed using samples from different preparations.

**Table 1:** Sequence of primer used.

S.NO	Primer	Primer Sequence	Accession Number
1	β-Actin	F: 5′-CGAGCAGGAGATGGGAACC-3′ R: 5′-CAACGGAAACGCTCATTGC-3′	AF057040
2.	iNOS	F: 5′-GGAGATGCAAGGTCAGCTTC-3′ R: 5′-GGCAAAGCTCAGTGACTTCC-3′	AY324390
3	TNF-α	F: 5′-ACCAGGCCTTTTCTTCAGGT-3′ R: 5′-TGCCCAGTCTGTCTCCTTCT-3′	AY427649
4	IL-1β	F: 5′-ATCCAAACGGATACGACCAG-3′ R: 5′-TCGGTGTCTTTCCTGTCCAT-3′	BC098597
5.	IL-4	F: 5′-AGTCACGCTGCTGATGAAGA-3′ R: 5′-AACTTGGTCTTGGGCTTTTT-3′	AB375404
6.	IL-10	F: 5′-ATAGGATGTTGCTGGGTTGG-3′ R: 5′-GTGGATGAAGTCCATTTGTGC-3′	AY887900
7.	TLR-4a	F: 5′-CGGCACTCCTCAAATCAACT-3′ R: 5′-GTCCTTCAAATCCTCCCACA-3′	NM _001131051
8.	IFN-γ	F: 5′-ATGATTGCGCAACACATGAT-3′ R: 5′-ATCTTTCAGGATTCGCAGGA-3′	AB158361

### JPtNPs as immunobooster for zebrafish

Immunobooster activity of JPtNPs was verified by resensitization experiment. For this experiment, about zebrafish was infected with *A. hydrophila* as mentioned above and then treated with 10 µl of 50 µM JPtNPs. All the fish survive the infection and lives normally under the laboratory conditions. After 21 days, the survived fish were injected only with 10 µl of 0.1 OD culture of *A. hydrophila*. On the regular interval (3, 6, and 12 h), fish were sacrificed and the total RNA was isolated from zebrafish using TRIzol. Gene expression studies were performed as described above and normalized to β-actin.

### Determination of antibody formation by radial immuno assay (RIA)

The formation of antibody in zebrafish following *A. hydrophila* infection was assessed by RIA. In a typical experiment, zebrafish from all the groups except Group A were collected after a period of 12 h and anaesthetized and sacrificed as mentioned earlier. Later blood was collected from the gills and tail region. Collected blood was allowed to coagulate at room temperature. After coagulation, the vials containing blood were spun at 3000 rpm for 10 min, 4 °C to separate the anti-serum. Heat killed *A. hydrophila* was used as antigen. RIA assay was performed in petri plates using 1% agarose dissolved in saline. The agar was dissolved by heating in microwave. After cooling the agarose to approx. 40 °C, heat killed *A. hydrophila* (100 µL from 0.1 OD culture) was added to the agarose solution and the agarose was immediately poured in petri plates and allowed to cool at room temperature. After cooling holes were made in the agarose and anti-serum (20 µL) obtained from different groups was then added to each well. The plates were incubated at room temperature for 24–48 h. To visualize the zone of equivalence the gel was stained with coommassie brilliant blue R-250 for 10 min followed by destaining for 4–5 h.

To analyze the immune boosting potential of JPtNPs, we further measured RIA by resensitization experiments. The experiment was designed by using infected zebrafish that had been previously treated with JPtNPs, as mentioned earlier. These fish were allowed to grow under normal conditions for 3 days and then reinfected with *A.hydrophila*. However, this time the fish was not treated with JPtNPs. After 3 h of infection anti-serum was collected and RIA was performed to detect the presence of antibodies in the fish blood. Similarly, in an extension of these experiments, another group of resensitized fish was reinfected with bacteria on day 7 and 21 and anti-serum used for RIA. These fish were never treated with JPtNPs over the course of the experiment.

## Conclusion

Do nanoparticles develop immune response to combat bacterial infection? In this study, we successfully treated *A. hydrophila* infected zebrafish with JPtNPs. Remarkably the rescued fish sustain repeated bacterial infection. Analyzing the expression of immune related genes revealed that JPtNPs is highly immunomodulatory. RIA suggests the possibility of bacteria specific antibody generation. Our results showed a newer mechanism of action of NPs against infection through modulating pro-inflammatory cytokines and also through promoting the production of bacteria specific antibodies. This study revealed that lectin functionalized nanoparticles has immense potential for the development of newer therapeutic modality to combat bacterial infection and also to prevent re-infection.

## Electronic supplementary material

Supplementary Information
