# Author Correction: Jacalin capped platinum nanoparticles confer persistent immunity against multiple *Aeromonas* infection in zebrafish

**DOI:** 10.1038/s41598-018-22719-6

**Published:** 2018-03-20

**Authors:** Khan Behlol Ayaz Ahmed, Thiagarajan Raman, Anbazhagan Veerappan

**Affiliations:** 1 School of Chemical and Biotechnology, SASTRA University, Thirumalaisamudram, Thanjavur, 613401 Tamil Nadu India; 2 Department of Advanced Zoology and Biotechnology, Ramakrishna Mission Vivekananda College, Mylapore, Chennai 600004 India

Correction to: *Scientific Reports* 10.1038/s41598-018-20627-3, published online 02 February 2018

The original version of this Article contained a typographical error in the spelling of the author Anbazhagan Veerappan, which was incorrectly given as Anbazhgan Veerappan. This has now been corrected in the PDF and HTML versions of the Article, and in the accompanying Supplementary Information file.

