# Supplementary material for: Jacalin capped platinum nanoparticles confer persistent immunity against multiple Aeromonas infection in zebrafish
Source: Sci Rep. 2018 Feb 2;8:2200. doi: 10.1038/s41598-018-20627-3 (PMC5797147; doi:10.1038/s41598-018-20627-3)
Supplement: Supplementary file 1 — Supplementary Information [file 41598_2018_20627_MOESM1_ESM.doc]

*Supporting information*

**Jacalin capped platinum nanoparticles confer persistent immunity against multiple *Aeromonas* infection in zebrafish**

Khan Behlol Ayaz Ahmed^a^, Thiagarajan Raman^a,b^*, Veerappan Anbazhagan^a^*

^a^School of Chemical and Biotechnology, SASTRA University, Thirumalaisamudram, Thanjavur – 613 401, Tamil Nadu, India

^b^Department of Advanced Zoology and Biotechnology, Ramakrishna Mission Vivekananda College, Mylapore, Chennai – 600004, India

*In vitro antibacterial activity of JPtNPs*

Minimum inhibitory concentration (MIC) was determined by resazurin microtiter (REMA) plate method. Briefly, 100 μL of PtNPs (0.5 mM) was added to the 96 well plate and serial diluted and the final volume was made to 100 μL using the LB medium. Next, 100 μL of suspension containing 1×10^5^ cfu/mL bacteria were added to the 96 well plates and allowed to grow at 30^o^C. After 24 h, 30 µL of a 0.01% (wt/vol) resazurin solution was added to each well and incubated for 2 h. The presence of viable cells was estimated by the colour change from blue to pink.

The antibacterial activity of JPtNPs was further tested by well diffusion method against the following pathogenic bacteria: *E. coli* (MTCC723)*, P. aeruginosa* (MTCC1688)*, S. aureus* (MTCC3160)*, B. subtilis* (MTCC441), *A.hydrophilia, V. cholerae* (MTCC3904*), K. Pneumoniae* (MTCC109), *B. thurengensis* (MTCC869), *S. typhi* (MTCC98), *Shigella* (MTCC1457), *P. vulgaris* (MTCC7299). Briefly, bacterial cultures were first grown aerobically at 37^o^C in Luria-Bertani (LB) media. The cultures were maintained by streaking on LB agar plates and incubated at 37^o^C for 24 h. After that, pure colonies were isolated and sub-cultured every fortnight. Each strain was swabbed uniformly onto the LB-agar plates using sterile cotton swabs. Wells of 10 mm diameter were made on LB-agar plates using gel puncture. Using a micropipette, a definite volume of nanoparticles solution (50 µM) was dispensed into each well on all plates. The zone of inhibition of bacteria was measured after incubation for 24 h at 37^o^C.

Bacterial count assay was performed in LB agar plate. Typically, agar in LB medium was autoclaved and cooled to around 60˚C. To this mixture, JPtNPs of defined concentration was added and poured into Petri plates and allowed to solidify at room temperature. After solidification, 0.1 OD_660_ *A. hydrophila* culture was swabbed on the LB-agar plate containing JPtNPs. The plates were incubated for 24 h at 37˚C. Control experiment was performed without JPtNPs. The experiment was performed in triplicates and the colonies were counted and reported in cfu unit.

*Hemocompatibility study*

To display the *in vivo* compatibility of PtNPs, its effect was studied on human red blood cells (RBCs). Briefly, 5 ml of blood in anticoagulant was obtained from a healthy volunteer. Blood was initially maintained at 4 ^o^C for 1h. Later blood was centrifuged at 2000 rpm for 10 minutes at 4 ^o^C, yellowish white colour plasma as the supernatant was removed and the pellet containing pure RBCs was resuspended in PBS and the centrifugation process was repeated two times to ensure complete removal of plasma. The blood was then finally suspended in PBS and stored at 4^o^C for further experiments. 4% blood in PBS was used for the hemocompatibility study. Two test tubes containing each of 5 ml blood was used. One test tube was added with 250 µM PtNPs and incubated at 4^o^C for 3 h. After 3 h, one drop of blood from both the test tube was placed on clean grease free slide and analysed using NIKON Eclipse Fluorescence Microscope using bright field mode.

*In vivo antibacterial activity of Jac-PtNPs*

Zebrafish (*Danio rerio*) irrespective of sex, 3-5 cm in length and weighing approx. 250-300 mg were purchased from the local market for this study. Zebrafish were handled according to Institutional Animal Care and Use Committee guidelines.The fishes were maintained in glass tanks containing tap water with continuous aeration at room temperature. Fishes were fed with commercial fish feed at regular intervals. Tank water was changed periodically, and water from the same source was used all the time. Tap water was first filtered and then allowed to stand at room temperature for 24 h before use. The fishes were acclimatized to laboratory conditions for a week before onset of experiments. All fish experiments were conducted as per institution animal ethics guidelines.

*Optimization of experimental dosages and conditions*

Optimization of bacterial dosage to zebrafish for establishment of infection was done by administering *A. hydrophilia* intramuscularly following Neeley et al., with minor modifications. Using a 3/10 –cc U-100 insulin syringe intramuscular injection was performed. The fish was initially anesthetized (150 mM MS-222) and then made to lie down on a wet sponge in a tank filled with water. The needle of syringe was inserted at an angel of 45˚ with the spine below the dorsal fin. 10µl of bacterial suspension was injected slowly. The control fishes were injected with equal volume of sterile PBS. The fishes were injected with different concentration of bacterial suspensions having an optical density in the range of 0.01, 0.05, 0.1, 0.3, and 0.5. The mortality of the fishes was observed for a period of 24 h. Depending upon the mortality data 0.1 OD bacterial culture sample was selected for further studies.


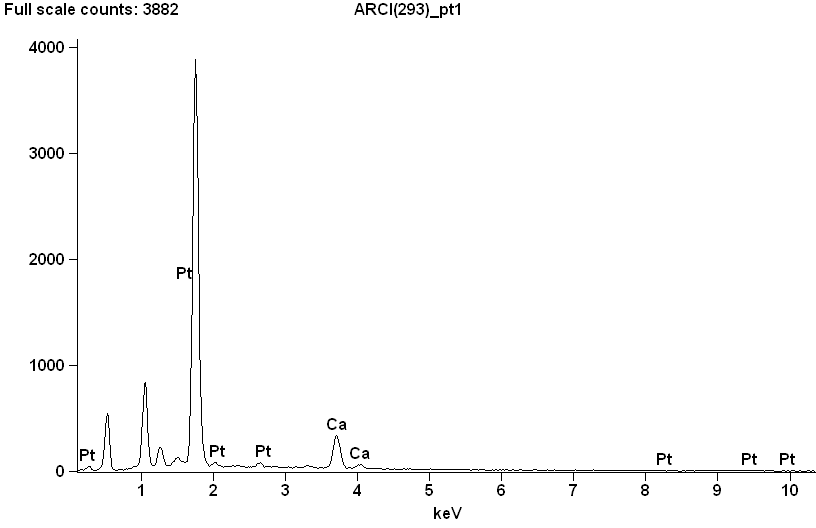


Fig. S1: EDAX analysis of JPtNPs shows the presence of elemental platinum.

Fig. S2: FTIR spectra of jacalin and JPtNPs. The broad band at 3438 cm^-1^ is attributed to –OH and –NH stretching vibrations of jacalin amino acids. A typical protein stretching modes of the –C=O, –CN and –NH bending are merged together and appeared as a single peak at 1602 cm^-1^. Amide III band corresponds to –CN stretching and –NH bending was observed at 1255 cm^-1^. The absorbance peak associated with the side chain vibrations was observed at 1358 cm^-1^. FTIR spectra of PtNPs display the all the peaks of jacalin, suggesting that the PtNPs is capped with jacalin.


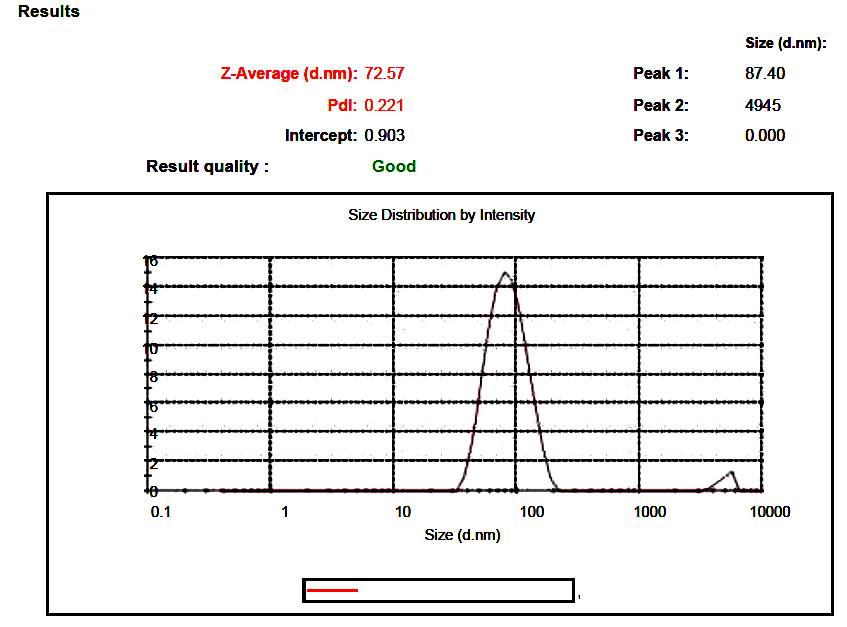


Fig. S3: Particle size analysis of JPtNPs. The average particle size is 72.57 nm with a polydispersity index (PDI) of 0.221. The lower PDI indicate that the nanoparticles are relatively monodispersed in the solution.


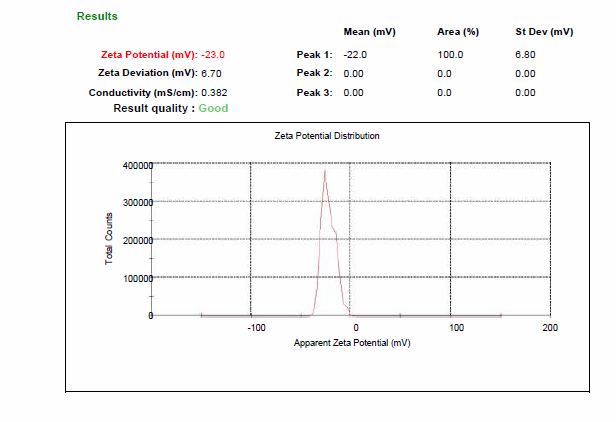


Fig. S4: Zeta potential of JPtNPs

Fig. S5: Determination of minimum inhibitory concentration using REMA. Briefly, 100 μL of JPtNPs (0.5 mM) was added to the 96 well plate and serial diluted and the final volume was made to 100 μL using the LB medium. Next, 100 μL of suspension containing 1×10^5^ cfu/mL *A. hydrophila* bacteria were added to the 96 well plates and allowed to grow at 37^o^C. After 24 h, 30 µL of a 0.01% (wt/vol) resazurin solution was added to each well and incubated for 2 h. The presence of viable cells was estimated by the color change from blue to pink. Blue color indicates dead cells and pink color indicate the presence of viable cells. The lowest concentration showing the blue color was recorded as MIC. Here it is 31.2 μM. Experiments were performed in tripilicate.


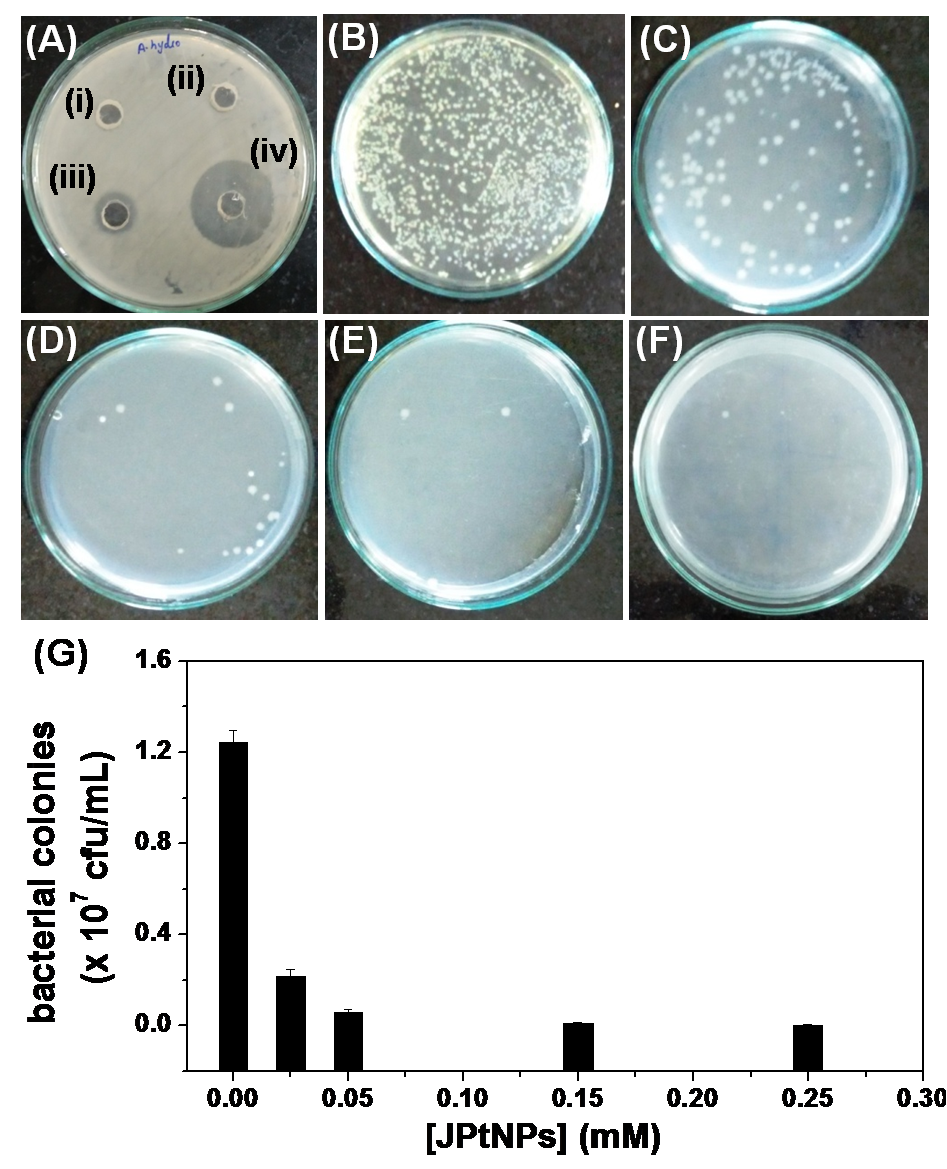


Fig. S6: *In vitro* antibacterial activity of JPtNPs against *A. hydrophilla*. (A) Zone of inhibition assay, (i) jacalin, (ii) chloroplatinic acid, (iii) 10 μL of 50 µM JPtNPs and (iv) 100 μL of 50 µM JPtNPs. (B)-(F) Bacterial colony count assay. Bacterial colonies formed on LB agar plates contains JPtNPs at the concentration of (B) 0 µM, (C) 25 µM, (D) 50 µM, (E) 150 µM and (F) 250 µM. (G) A plot of number of viable bacteria remains in the 24 h incubation samples. Data were counted from the CFU results in (B)-(F).

Fig. S7: Antibacterial activity of JPtNPs against Gram-positive and Gram-negative organism. Zone of inhibition at different volume of 50 µM JPtNPs. (A) *P. aeroginosa,* (B) *S. aureus,* (C) *V. Cholerae,* (D) *K. Pneumoniae,* (E) *B. Thurengensis,* (F) *E. coli,* (G) *S. typhi,* (H) *Shigella,* (I*) P. vulgaris,* (J) *B. Subtilis and* (K) measured ZOI in mm. In each panel (i) jacalin, (ii) hexachloroplatinic acid, (iii) 10 μL of 50 µM JPtNPs (in panel K, black bar)and (iv) 100 μL of 50 µM JPtNPs (in panel K, gray bar). Noteworthy, jacalin and hexachloroplatinic acid has no ZOI.

Fig. S8: Bacterial colony count assay. Bacterial colonies formed on LB agar plates contains 0.1 mM of pectin capped PtNPs (pPtNPs), mixture of jacalin and pPtNPs (JpPtNPs), and jacalin capped PtNPs (JPtNPs). As noted from the figure the presence of jacalin improves the antibacterial activity of the NPs.

Fig. S9: Hemocompatibility assay. (A) Untreated human RBC and (B) human RBC treated with 250 µM JPtNPs. About 5 ml of blood in anticoagulant was obtained from a healthy volunteer. Blood was initially maintained at 4^o^C for 1 h. Later blood was centrifuged at 2000 rpm for 10 minutes at 4^o^C, yellowish white colour plasma as the supernatant was removed and the pellet containing pure RBCs was resuspended in PBS and the centrifugation process was repeated two times to ensure complete removal of plasma. The blood was then finally suspended in PBS and stored at 4^o^C for further experiments. 4% blood in PBS was used for the hemocompatibility study. Two test tubes containing each of 5 ml blood was used. One test tube was added with 250 µM JPtNPs and incubated at 37^o^C for 3 h. After 3 h, one drop of blood from both the test tube was placed on clean grease free slide and analysed using NIKON Eclipse Fluorescence Microscope using bright field mode.

Fig. S10: *In vivo* antibacterial activity. (A) infected group (B) infected fish treated after 3 h with 10 μL of 0.05 mM JPtNPs
